# Editorial: Beneficial Microbes Alleviate Climatic Stresses in Plants

**DOI:** 10.3389/fpls.2019.00595

**Published:** 2019-05-16

**Authors:** Ying Ma, Miroslav Vosátka, Helena Freitas

**Affiliations:** ^1^Department of Life Sciences, Centre for Functional Ecology-Science for People and The Planet, University of Coimbra, Coimbra, Portugal; ^2^Institute of Botany, Academy of Sciences of the Czech Republic, Pruhonice, Czechia; ^3^Department of Experimental Plant Biology, Faculty of Science, Charles University, Prague, Czechia

**Keywords:** plant growth promoting microorganisms, biotechnology, climate change induced-stresses, sustainable agriculture, environmental decontamination

Global climate change accelerates the concurrence of a variety of abiotic (e.g., drought, salinity, heavy metals, and extreme temperatures) and biotic stresses (e.g., phytopathogens), thus considerably affecting agricultural productivity and bioremediation efficiency, even forest ecosystems. In this scenario, plant growth promoting microorganisms (PGPM) are receiving increasing attention of agronomists and environmentalists as candidates to develop an effective, eco-friendly, and sustainable alternative to conventional agricultural (e.g., chemical fertilizers and pesticide) and remediation (e.g., chelators-enhanced phytoremediation) methods employed to deal with these climate change-induced stresses (Ma et al., 2011, 2016, Ma et al.). Research on PGPM [e.g. (plant growth promoting bacteria (PGPB), rhizobia, arbuscular mycorrhizal fungi (AMF)] have shown great potential in the management of various agricultural and environmental problems, however, to date, a collective database on the role of PGPM in alleviating various climatic stresses in plants is not available. Therefore, this research topic was launched to advance the knowledge of the mechanisms underlying plant-microbe interactions, review recent progress and address some of the challenges, thus providing the opportunities to translate basic knowledge into sustainable applications.

Salinity is a major abiotic stress limiting the growth and productivity of plants worldwide. It has been proved that harnessing the potential of plant growth promoting rhizobacteria (PGPR) is an alternative strategy to improve plant stress tolerance. Ilangumaran and Smith provide a comprehensive review of major research advances on physiological, biochemical, and molecular mechanisms attributed by PGPR regulating plant adaptation and tolerance to salinity stress. They summed up the principal mechanisms including (1) improvement of water and nutrient uptake, photosynthesis, and source-sink relationships; (2) activation of antioxidant activity, osmolyte accumulation, proton transport machinery, salt compartmentalization, and nutrient status; (3) modulation of phytohormone status, gene expression, protein function, metabolite synthesis, and secretion of signaling molecules and initiation of stress-responsive pathways. The systems biology perspective on plant-microbe interactions in response to salinity opens up new prospects of understanding the regulatory networks of PGPR induced plant salt tolerance, suggesting application of PGPR could serve as a promising measure to alleviate salt stress and improve global food production.

Among the mechanisms involved in plant-microbe interactions, microbial phytohormone production plays a crucial role in the stimulation of plant defense response against abiotic stress. A comprehensive review of the function, aspects and production of microbial phytohormones [e.g., auxins, cytokinins, abscisic acid (ABA), gibberellic acid, and salicylic acid] involved in the improvement of abiotic stress tolerance and defense response in crop plants under hostile environments (e.g., drought, salinity, nutrient deficiency, or heavy metal contamination) was provided by Egamberdieva et al.

As an important component of global climate change, increasing nitrogen (N) deposition and its ecological consequences are of serious concern, since it may profoundly influence the structure and function of the forest ecosystem. Zhao et al. performed a field trial in a mixed deciduous forest of China with canopy addition of N and water for 4 years and found that the effect of increased N deposition and precipitation on AMF community composition was time-dependent, mediated by soil factors, and possibly related to the sensitivity and resilience of forest ecosystem to global changes.

The further understanding of how successful PGPR colonization can improve the growth and defense response of the plants against fungal pathogens under salinity stress implies the diverse physiological and biochemical mechanisms used by PGPR to confer both abiotic and biotic stress tolerance of host plants. This was what Jha and Singh exemplified to us in a study of the potential of PGPR *Stenotrophomonas maltophilia* SBP-9 to promote the growth of *Triticum aestivum* under biotic (*Fusarium graminearum*) and abiotic (salt) stresses.

Additionally, Egamberdieva et al. described a detailed study of the functional role of endophytic PGPB colonizing root tissues in enhancing the growth performance and controlling root rot in *Cicer arietinum* under saline soil conditions. They demonstrated the importance of endophytic bacterial colonization for the improved symbiotic performance of legume host plants with rhizobia, with increased root growth, nutrient bioavailability and faster osmotic adjustment, and reduction in H_2_O_2_ production and pathogen infection under saline soil conditions.

The emission of volatile organic substances (VOCs) of microorganism can increase disease resistance and abiotic stress tolerance, and thereby helping plants control a wide range of pathogens. Notably, Rybakova et al. combined *in vitro* and in planta methods with the study of the mode of interaction between PGPB and phytopathogens via their VOCs. They highlighted the identification of several antimicrobial and plant growth promoting VOCs and several other antimicrobial volatile substances that were produced by both *Paenibacillus polymyxa* and *Verticillium longisporum* as a reaction to one another's VOCs, and the regulation of their general metabolic activities. The findings contribute to a better understanding of the mechanisms of VOCs underlying the interaction between pathogens and their natural antagonists.

Common mycorrhizal networks (CMNs) by AMF interconnect plants, which play a crucial role in redistributing nutrients and draining carbon (C) from the different plant partners at different rates (Walder and van der Heijden, 2015). Rezáčová et al. established an intercropping experiment of two similar *Panicum* species with contrasting carbon (C_3_ and C_4_) metabolism. The plants were subjected to two different temperature regimes and inoculated with or without a consortium of AMF. A root-free compartment (RFC), but AMF-accessible, was added that was comprised of ^15^N-labeled clover residues, which could allow direct assessment of the benefit of AMF on the plants in term of N derived from the organic biomass. Also, the isotopic C signature of the plants enabled the determination of which plant species were the main donor of C to the AMF. The authors found that the C_3_ species continued feeding the CMNs by using the specific C signature of the plant, AMF biomass/specific lipids in the root compartments and in the RFC. Other important results are that the inoculation of AMF reduced the plant biomass production, but increased plant phosphorus uptake under both temperature regimes.

One example of novel applications for the sustainable use of diazotrophic bacterial and yeast endophytes in modulating stomatal behavior and increasing plant water relations was shown by Rho et al. The authors analyzed the effects of endophyte inoculation (multiple or single strain) on leaf water potential, whole-plant water use, and stomatal responses of *Oryza sativa* under CO_2_ enrichment and water deficit. They found that the ABA production by endophytes contributed significantly to the stomatal reactions and the resulting plant physiological benefits.

Scientists from different fields of research, from basic science to applied science, are indeed contributing to the understanding of the molecular, cellular and physicochemical mechanisms underlying plant-microbe interactions under various environmental stresses, which will certainly contribute to generating novel solutions for the development of PGPM-based sustainable applications in agricultural and forestry systems.

## Author Contributions

YM draft the editorial text. YM, MV, and HF revised and approved the final version of the editorial text.

### Conflict of Interest Statement

The authors declare that the research was conducted in the absence of any commercial or financial relationships that could be construed as a potential conflict of interest.
